# Meningitis by Streptococcus suis: Case Report of a Critically Ill Patient

**DOI:** 10.7759/cureus.73044

**Published:** 2024-11-05

**Authors:** Joana Nogueira, Leonor Simões, Emília Trigo, José Eduardo Mateus, Ricardo Freitas

**Affiliations:** 1 Intensive Care Unit, Centro Hospitalar e Universitário de Coimbra, Unidade Local de Saúde (ULS) de Coimbra, Coimbra, PRT

**Keywords:** critical care, meningitis, occupational disease, streptococcus suis, zoonotic pathogen

## Abstract

*Streptococcus suis* is a zoonotic pathogen commonly found in pigs, capable of causing disease in humans when in contact with contaminated pigs or pork products. The most common presentation in humans is meningitis, although it can also manifest as sepsis, arthritis, endocarditis, or endophthalmitis. In Europe, most cases are described as occupational disease related to pig farming or raw pork handling. We report a severe case of meningitis by *S. suis* in a 51-year-old man who worked as a piglet roaster. The patient presented to the emergency department in a comatose state (Glasgow Coma Scale of 7) after experiencing headache, fever and generalised myalgia. No focal or lateralizing motor signs were found on the neurological examination. The brain computed tomography scan revealed diffuse cerebral oedema and excluded other associated vascular or parenchymal lesions. Bacterial meningitis was confirmed after cerebrospinal fluid examination and empiric antibiotic therapy was started. This critically ill patient presented a rapid and fatal progression of the disease. We aim to describe a fulminant presentation of meningitis by *S. suis *and emphasize the importance of recognizing and promptly treating this condition, particularly in high-risk populations.

## Introduction

Bacterial meningitis is commonly associated with infection by *Streptococcus pneumoniae*, *Neisseria meningitidis*, *Haemophilus influenzae*, and *Listeria monocytogenes*, when acquired in the community [1]. However, other pathogens should also be considered.

*Streptococcus suis* is a facultatively anaerobic gram-positive bacteria, commonly present in the upper respiratory tract of pigs. It is an emerging zoonotic agent that can cause disease in both pigs and humans, usually manifesting as meningitis. However, it can also manifest as sepsis, arthritis, endocarditis, or endophthalmitis. In humans, infection with this zoonotic pathogen can occur through the consumption of raw, contaminated pork meat or direct contact with infected pigs. Transmission typically occurs through the contamination of skin abrasions or from pig bites [2].

In Europe, meningitis by *S. suis* is usually an occupational disease in individuals working in pig farming or manipulating raw pork meat [3]. Patients may progress to a severe presentation and mortality in *S. suis* meningitis can be up to 12.8% [4]. Therefore, early recognition of this disease in high-risk groups and awareness of its clinical course is important, as this might impact the patient’s treatment and prognosis.

Herein, we describe the diagnosis, treatment and clinical progression of a critically ill patient with a fulminant presentation of meningitis by *S. suis*.

## Case presentation

A 51-year-old male patient was admitted to the emergency department due to impaired consciousness, Glasgow Coma Scale of 7 (E2 M4 V1). Additionally, the patient presented with headache and generalised myalgia for the last two days and altered mental status for the last eight hours. The patient did not experience nausea or vomiting.

According to his personal medical history, he was a smoker and had a trauma in his right ear canal a year ago. There were no chronic medication prescriptions. His occupation was roasting piglets at a local restaurant, a common activity in his residential and working area due to traditional gastronomy.

At admission, the patient presented with isochoric and isoreactive pupils but with ocular deviation to the right, nuchal rigidity, and tonic posture of the upper limbs without motor lateralization or pathological reflexes. He was hemodynamically stable, had an ear temperature of 38.4ºC, and capillary blood glucose was 228 mg/dL.

Orotracheal intubation was performed due to the patient’s impaired consciousness with rapid sequence induction (propofol, fentanyl, and rocuronium). Brain computed tomography (CT) scan and CT angiography (CTA) revealed diffuse cerebral oedema with no other vascular or parenchymal lesions associated, which excluded ischemic or hemorrhagic stroke and space-occupying lesions. Serologic laboratory tests showed a leucocytosis of 15.2 x 10^9^/L and neutrophilia (13.98 x 10^9^/L, 91.9%).

A lumbar puncture was performed approximately 40 minutes after the patient’s admission to the emergency room and after performing the CT scan. The cerebrospinal fluid (CSF) was purulent and had a quick drip, suggesting a high opening pressure. Its analysis, presented in Table 1, revealed high protein levels, extreme glucopenia, elevated lactate levels and 18930 leukocytes/mm^3^, with a predominance of polymorphonuclear cells (98%).

**Table 1:** Cerebrospinal fluid analysis

Parameters	Value	Normal range
Protein	636 mg/dL	15 -40 mg/dL
Glucose	< 5 mg/dL	40-70 mg/dL
Lactate	15,12 mmol/L	< 2 mmol/L
Leukocytes	1890/mm^3^	< 3/mm^3^
Erythrocytes	< 3/mm^3^	< 3/mm^3^

Biological samples were collected for microbiology investigations from blood, CSF, tracheobronchial secretions, and urine. As part of the microbiology investigation, CSF was also collected to be analysed with the FilmArray® meningitis/encephalitis panel (BioFire Diagnostics, Salt Lake City, Utah, United States).

Considering the clinical presentation and the changes in the CSF, empiric antibiotic therapy for bacterial meningitis was started in the emergency room 50 minutes after admission with ceftriaxone 2g, vancomycin 2g, and ampicillin 2g, and the patient was admitted to ICU two hours after hospital admission. No microorganisms were found from the FilmArray meningitis/encephalitis panel, so empiric treatment with acyclovir was initiated, considering the patient’s critical state. Therapy with dexamethasone 12 mg every six hours was also introduced.

**Figure 1:**
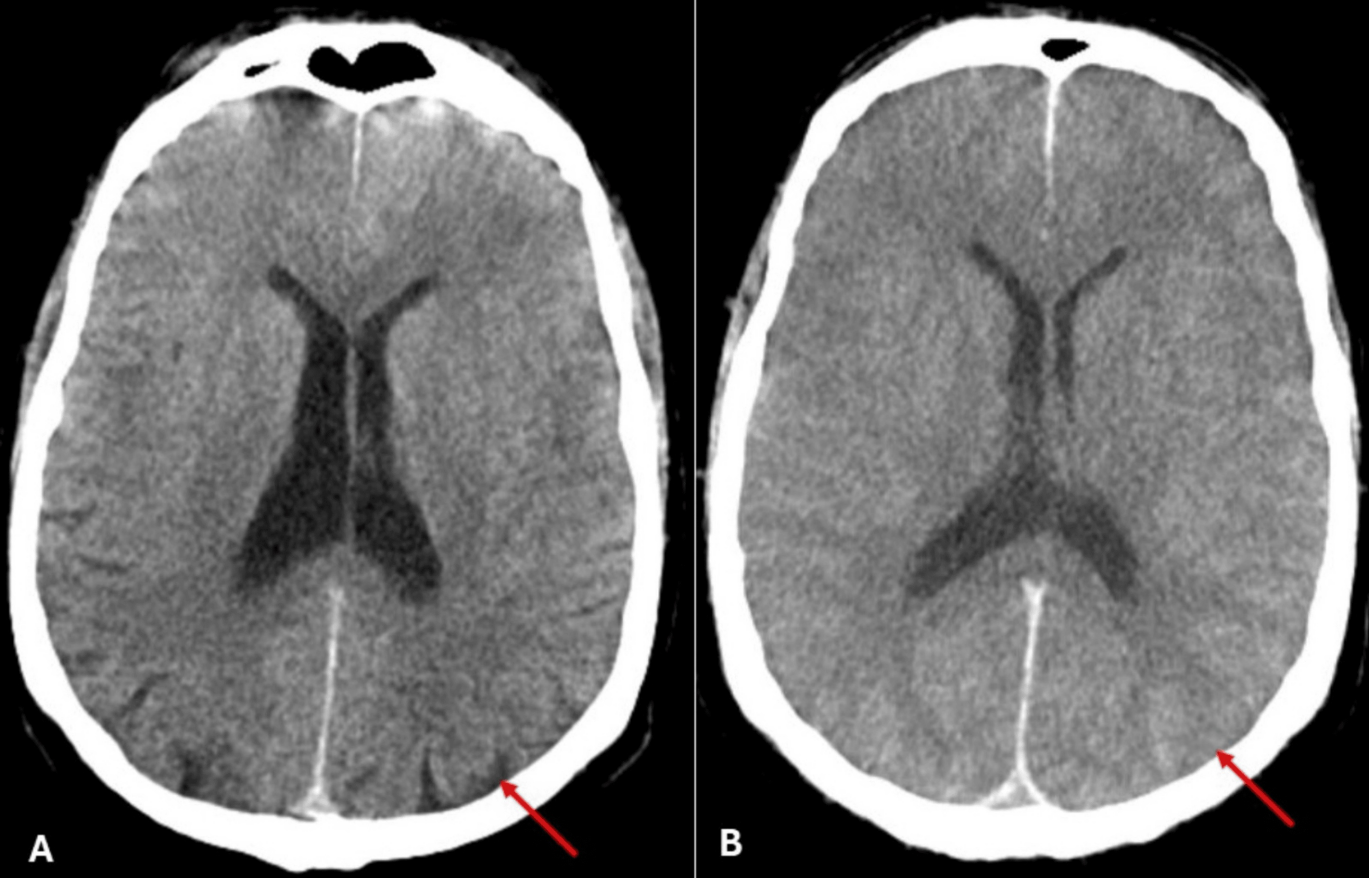
Comparison between non-contrast brain CT at admission (A) and at the 20-hour revaluation (B). (A) Admission CT showing diffuse cerebral oedema, but with preservation of brain sulci and gyri (red arrow); (B) Revaluation CT showing worsening of diffuse cerebral oedema, with effacement of the brain sulci and gyri and with loss of grey and white matter differentiation (red arrow). CT: computed tomography

Twenty hours after hospital admission, a neurosurgical consultation was obtained and an invasive intracranial pressure sensor was placed in the right frontal cranial area, demonstrating severe intracranial hypertension (83 mmHg). The pupils were mydriatic and non-reactive to light, and the patient developed central diabetes insipidus. A revaluation brain CT scan was performed to define the aetiology of intracranial hypertension. Compared to the admission CT, it revealed worsening of the cerebral oedema (Figure 1) and the cerebral tonsils extending beyond the magnum hole.

After a multidisciplinary discussion, no additional surgical or medical therapies were contemplated due to the patient’s severe brain oedema and clinical evidence of vital poor prognosis.

On the second day of hospital stay, *S. suis* was found in the CSF sample as the infectious pathogen. It was susceptible to all tested antibiotics, except for erythromycin and clindamycin. The strain was sensible to β-lactam antibiotics. The laboratory was not able to test and determine the serotype. Three days after hospital admission, brain death was declared. Due to his epidemiological history of contact with piglets, the public health authorities were notified.

## Discussion

*S. suis* is a zoonotic pathogen known to colonise the upper respiratory tract in pigs and can cause disease in both pigs and humans. The most common presentation of infection in humans is meningitis (68% of cases), followed by sepsis, arthritis, endocarditis, and endophthalmitis [4].

In certain East and Southeast Asian countries such as Vietnam, Thailand, and China, *S. suis* is among the more common pathogens responsible for meningitis. The local population in these countries is at risk due to habits of consuming raw or undercooked pork and inadequate production and slaughtering practices [2].

Approximately 10% of reported cases of infection by *S. suis* are from Europe [5], and are usually described as an occupational disease in individuals in contact with infected pigs or contaminated meat, mostly through skin abrasions. The population at risk are butchers, hunters, slaughterhouse workers, lorry drivers, and meat factory workers [3].

Reports from a study in the Netherlands described an estimated annual risk of developing *S. suis* meningitis in pig breeders and abattoir workers of 3.0/100000, a rate 1500 times higher than the general population [6]. A recent European report also showed an incidence of 0.161-4.945 cases/100000 persons in the risk population [3]. The patient, in this case report, was part of the high-risk group for meningitis by this zoonotic pathogen because he manipulated carcasses of piglets to roast them. It is also known that the highest rate of colonisation and disease in pigs usually occurs in piglets between 4-10 weeks of age [2], which might have contributed to a higher exposing risk in this patient.

Differing from other European countries, there have been few case reports of meningitis by *S. suis *in Portugal, suggesting that this zoonotic disease might be underdiagnosed in this country [7].

Most strains of *S. suis* have a high susceptibility to β-lactam antibiotic [8] so, usually, the empiric antibiotherapy for community-acquired meningitis is adequate for this microorganism. In our clinical case, even though the *S. suis* strain was susceptible to the administered antibiotics, the patient's initial presentation was severe, marked with extensive cerebral oedema, and the patient quickly evolved to death a few days after the first symptoms. A meta-analysis described a case-fatality rate of 12.8% in humans with infection by *S. suis*, and permanent sequelae including hearing loss (39.1%) and vestibular dysfunction (22.7%) [4].

When patients present *S, suis* severe meningitis, other treatment modalities beyond antibiotics should be considered, such as vital support measures and anti-oedema therapy. Clinicians should be aware of the need for early detection of extensive cerebral oedema and intracranial hypertension. Invasive intracranial pressure monitoring and non-invasive methods (cerebral ecodoppler or ecographic optic nerve sheath measurements) could be combined with serial CT scans for this purpose. A multidisciplinary approach is also essential for improving patient outcomes and treatment success in these cases.

Clinical case reporting to health authorities is crucial for implementing preventive measures in pig farming. Health professionals should be vigilant in recognizing this zoonotic pathogen to collect important epidemiological data and give preventive recommendations to high-risk groups.

## Conclusions

*S. suis *meningitis is considered an occupational disease in Europe, with potentially detrimental outcomes for patients leading to critical illness. Given its preventable nature, reporting clinical cases to authorities and implementing protective measures among high-risk groups could have a significant impact.

Healthcare professionals need to be vigilant of this emerging zoonotic disease, as it may be underdiagnosed in various countries. Meningitis by* S. suis *should be considered among individuals working in pig farming or manipulating raw pork meat who present with meningitis. A multidisciplinary approach is important when facing severe meningitis by *S. suis*, and there should be a raised awareness for early detection and treatment of extensive cerebral oedema and intracranial hypertension.
